# The consequences of ultra-processed foods on brain development during prenatal, adolescent and adult stages

**DOI:** 10.3389/fpubh.2025.1590083

**Published:** 2025-06-23

**Authors:** Gaia Mottis, Pratheba Kandasamey, Daria Peleg-Raibstein

**Affiliations:** ^1^Department of Health Sciences and Technology, ETH Zurich, Zurich, Switzerland; ^2^Institute for Neuroscience, Department of Health Sciences and Technology, ETH Zurich, Zurich, Switzerland; ^3^Institute of Food, Nutrition and Health, Department of Health Sciences and Technology, ETH Zurich, Zurich, Switzerland; ^4^Neuroscience Center Zurich, University of Zurich, ETH Zurich, Zurich, Switzerland

**Keywords:** ultra processed food, maternal, nutrition, offspring, cognition, eating behaviors, motivation, brain development

## Abstract

Ultra-processed foods (UPFs) are highly refined formulations of non-nutritive compounds containing elevated amounts of sugar, fat, sodium, food additives, and dietary emulsifiers. Consumption of UPF is robustly linked to a range of non-communicable diseases, including obesity, type 2 diabetes, cardiovascular disease, and mental disorders in adults. While substantial research highlights the negative health impacts of UPFs in adulthood, their effects on brain development during critical periods of biological vulnerability, pregnancy, childhood, and adolescence, remain underexplored. During pregnancy, significant metabolic and physiological adaptations occur to support fetal growth, making maternal diet quality essential for optimal perinatal outcomes. Poor maternal nutrition, including high UPF consumption, has been associated with an increased risk of hypertensive disorders, gestational diabetes, and adverse birth weights, potentially predisposing offspring to metabolic health disorders later in life. Similarly, in early childhood, inadequate nutrition is a key risk factor for developmental impairments, influencing cognitive function and long-term health outcomes. Adolescence, another critical stage of brain maturation, is particularly susceptible to the effects of micronutrient deficiencies, often exacerbated by diets high in UPFs, which can impair neurodevelopment and cognitive performance. As UPFs continue to dominate modern diets, accounting for over 50% of total energy intake in some developed nations, understanding their long-term impact on brain development is crucial. Early-life exposure to UPFs may contribute to lasting cognitive deficits and increased susceptibility to mental health disorders, emphasizing the urgent need for targeted dietary interventions and public health strategies aimed at pregnant women, children, and adolescents.

## Introduction

For several years, researchers and medical experts have increasingly investigating the role of ultra-processed foods (UPF) in relation to quality, quantity, and impact in human health. Numerous studies have investigated how UPF consumption may contribute to weight gain, obesity, metabolic health disorders, depression, attention deficit hyperactivity disorder (ADHD), and other gut and brain health conditions (1–3), as well as its potential impact on mental well-being (3, 4).

A growing body of evidence strongly suggests that the global rise in obesity parallels the increasing availability and consumption of calorie-dense, nutrient-poor UPFs, implicating them as a key driver of the obesity epidemic (5). However, the impact of UPFs extends beyond metabolic health alone. This correlation highlights the urgent need to assess the broader implications of UPF consumption, including its influence on neurological function, cognitive development, and mental health.

To fully grasp the public health implications of UPFs, it is crucial to examine the various factors that have driven their widespread consumption in recent decades. Researchers have explored large-scale economic transformations that have expanded the production and accessibility of these highly processed foods (6, 7), the shifting dietary patterns observed across diverse global populations (8) and the complex psychological mechanisms that shape food preferences and decision-making (9).

Researchers are concerned about the potentially toxic and addictive elements of UPFs and their brain-based impacts. Furthermore, research suggests that UPF consumption has increased in popularity worldwide (10). This coincides with the global increase in obesity and mental ill health. Their widespread appeal and growing dominance as a primary calorie source raise urgent questions about the role of food systems in shaping health outcomes. The continuous rise in obesity, diet-related non-communicable diseases, mental health disorders, and neurodegenerative conditions cannot be attributed solely to genetic factors or an aging population (4). Could it be that decades of UPF consumption have contributed to an escalating cycle of health deterioration, beginning with the youngest and most vulnerable populations?

Notably, exposure to UPFs now begins before birth, as an increasing number of women of childbearing age consume these foods prior to conception, during pregnancy, and throughout breastfeeding (11–15). This early nutritional environment may have lifelong metabolic and neurodevelopmental consequences, reinforcing an intergenerational cycle of poor health outcomes. In light of these challenges, understanding the underlying drivers of UPF consumption and their long-term effects is essential for developing effective strategies to mitigate their impact on global health.

This review examines the effects of UPF consumption on brain development and function across different life stages, from maternal pregnancy through childhood, adolescence, and adulthood. Specifically, we will identify critical periods of brain development, the brain regions involved in regulating eating behaviors, and the biochemical mechanisms through which UPFs may disrupt neurological function. By synthesizing current evidence, this work aims to provide a comprehensive understanding of the potential neurodevelopmental and cognitive risks associated with UPFs, emphasizing the importance of dietary interventions for long-term health.

## The rise of ultra-processed foods: changing modern diets

Discussions around highly processed foods began in the 1980s, particularly regarding the rise of convenience foods and products high in synthetic additives (10). However, the roots of ultra-processed food consumption trace back earlier. While frozen meals were developed as early as the 1940s for use in airlines and institutions, it was C. A. Swanson and Sons who popularized the concept for home consumers in 1953 with the launch of the “TV dinner” (16). This innovation, designed to align with the postwar rise of television culture, marked a turning point in the commercialization of convenience foods. The spread of household microwave ovens starting in 1967 further accelerated the appeal and accessibility of these meals. These developments laid the foundation for later critiques, emerging in the 1980s, about the health and environmental impacts of industrially processed foods. These early warnings were part of broader movements focused on dietary health and environmental sustainability. However, the specific term “ultra-processed foods” was not introduced until 2009, when Carlos Monteiro developed the NOVA classification system to categorize foods based on the extent and purpose of their processing (17). Since then, debates over the health impact of UPFs have intensified, with a growing body of evidence linking their consumption to poor diet quality, metabolic disorders, and non-communicable diseases (18, 19).

In recent years, scientific advancements and heightened public awareness have fueled renewed interest in the potential health risks associated with UPFs (20). This scrutiny extends into the food processing industry, which plays a crucial role in shaping modern diets. Food processing aims to meet consumer demands by extending shelf-life, enhancing taste, ensuring microbiological safety, and improving affordability and accessibility (21). However, concerns persist regarding the negative impact of these practices on dietary quality, particularly in relation to nutrient density, food additives, and metabolic health effects (22, 23).

To address these challenges, the NOVA classification system was developed, providing a structured framework for distinguishing UPFs from minimally processed and whole foods. According to Monteiro, “*ultra-processed foods are formulations of ingredients, mostly of exclusive industrial use, that result from a series of industrial processes*” designed to create highly palatable, convenient, and marketable products. This classification has since been widely adopted in nutrition research to examine the health implications of UPF consumption. While various terms have been used to describe highly palatable or rewarding foods (24, 25), no universal terminology has been established due to ongoing debate in the field (26, 27). To ensure consistency, we use the term “ultra-processed foods” (UPFs) in alignment with the widely recognized NOVA system. These products are characterized by high energy density, containing large amounts of saturated and trans fats, salt and refined sugars. At the same time, they are poor sources of essential nutrients, including dietary fibers, protein, vitamins and minerals. Furthermore, UPFs typically contain additives, such as colorants, flavor enhancers, emulsifying salts and sweeteners, to improve their palatability and make them highly attractive. Research has also frequently highlighted the presence of harmful compounds derived from processing and packaging practices (28, 29).

The NOVA food classification, which is the most currently and widely used by researchers, categorizes food into four main groups based on their extent of processing (17):

1. Unprocessed and minimally processed foods such as fruit and vegetables, milk, eggs and meats.
2. Processed culinary ingredients, including oils, butter, lard, sugar and salt.
3. Processed foods such as canned fish, legumes or cheeses, usually adding salt, oil, sugar and using preservation methods such as canning and bottling.
4. Ultra processed foods (UPFs).

Over the past decades, the consumption of UPFs has increased, currently accounting for a dominant portion of the global diet (30–32). Particularly, UPFs represent the highest percentage of total dietary energy intake in developed countries, including the USA, Canada and the UK, with their consumption also rapidly growing in middle-income nations (1, 10, 32, 33). This trend has raised widespread concern in the scientific and medical field and is the subject of numerous studies aiming to better understand the effects of these foods on human health. The growing consumption of UPFs among children and adolescents is alarming. This trend poses a threat to their development, especially of the brain and nervous system, and may lead to various neurological and mental disorders, eating disorders and other issues (1, 34–37).

## Health consequences and outcomes linked to UPFs

Epidemiological studies have consistently demonstrated that dietary risk factors, specifically exposure to certain foods and dietary patterns, significantly contribute to the global burden of disease (34). More specifically, UPF consumption was associated with increased body mass index (BMI), weight gain and prevalence of obesity in both children and adults (1). In addition, it was linked to several cardio-metabolic outcomes, such as hypertension, metabolic syndrome and elevated cholesterol levels leading to an overall increased risk of type 2 diabetes, cardiovascular diseases and some types of cancer (1, 38). More recently, these food products have been implicated in higher rates of mental disorders such as depression and anxiety, as well as inattention and hyperactivity, especially in the younger population (1, 3, 36, 37). Building on this body of evidence, a systematic review and meta-analysis focusing on the period of pregnancy has reiterated the intake of UPFs as an indicator of poor diet quality, which increases the risk of gestational diabetes and preeclampsia (12). Furthermore, inadequate eating habits during pregnancy have been associated with the maternal development of chronic diseases later in life, such as diabetes mellitus, obesity, hypertension and cardiovascular disease. Some individual studies included in the review have also linked the consumption of UPFs to adverse outcomes in the newborn, such as preterm birth and congenital heart defects (12).

In addition to the development of serious diseases, the reduced nutritional intake of minerals and vitamins from UPFs, and thus the resulting unbalanced meals, leads to micronutrient deficiencies, which are even more essential during the growth phase of children.

## Socioeconomic, lifestyle, and psychological factors driving UPF consumption

The increasing prevalence of UPF consumption is influenced by multiple interrelated factors. Socioeconomic status plays a crucial role in shaping dietary habits among adults, which in turn impacts children and adolescents. Lower educational levels and limited economic resources are often associated with less diverse, lower-quality diets, leading to a higher reliance on affordable, energy-dense UPFs. These products are particularly appealing due to their low cost, easy accessibility, and minimal preparation time, making them a convenient choice for individuals with financial or time constraints (3, 39). This is especially true in households led by working mothers, single parents, or blended families, where time and energy for home-cooked meals may be limited. Studies have shown that adolescents in single-mother families are more likely to have unhealthy eating habits compared to those living with both parents (40). Additionally, families with unemployed parents or single-parent households are disproportionately likely to be high UPF consumers (39, 41, 42). Moreover, school meal programs, which represent a significant portion of children’s daily nutrition, vary widely in quality across regions. In many cases, meals provided in educational settings are high in ultra-processed items, reinforcing poor dietary habits from a young age. For instance, a study in the UK found that 64% of the calories consumed in school meals were from UPFs. Another study highlighted that, on average, UPF intake was high in both primary (72.6% of total lunch kcal) and secondary schoolchildren (77.8% of total lunch kcal) (43, 44). Beyond economic factors, modern lifestyle habits such as urbanization, frequent snacking, dining out, and poor sleep quality have all been identified as key drivers of UPF exposure (45).

Urbanization, frequent snacking, dining out, and poor sleep quality have all been identified as key drivers of UPF exposure (41). Additionally, the school environment plays a pivotal role in shaping eating behaviors among children and adolescents, influencing both food availability and dietary preferences (46). Adolescents, in particular, are among the highest consumers of UPFs, with studies estimating that 29 to 68% of their total energy intake comes from these products (21, 47). This trend is evident in broader dietary shifts, where the proportion of UPFs in total food purchases nearly tripled between 1990 and 2010, increasing from 11.0 to 32% (48).

Age also plays a significant role in UPF consumption patterns, exhibiting an inverse relationship with intake levels. Younger adults, adolescents, and children consume the highest amounts of UPFs (49), with consumption declining as age increases. Several other sociodemographic factors also contribute to differences in UPF intake, including race/ethnicity, income level, country of birth, geographic region, urban or rural residence, and food insecurity. However, cultural context significantly shapes dietary habits. For example, Japan provides a noteworthy contrast: school meals are considered part of food education (shokuiku) and are typically prepared from scratch using fresh, seasonal, and minimally processed ingredients. Meals are designed to be nutritionally balanced and are eaten in a communal setting with an emphasis on manners and appreciation for food. This national approach has contributed to low childhood obesity rates and reduced reliance on ultra-processed foods in school environments, distinguishing Japan from many Western countries where school meals often include a high proportion of UPFs (50, 51).

Individuals residing in urban areas and those who were unmarried, single, separated, or divorced tend to have higher UPF consumption, while the impact of education, income, and socioeconomic status on UPF intake varies across different countries. Urban living, in particular, has been identified as a key predictor of greater UPF consumption, aligning with global trends in UPF sales and household purchases (8, 52). Additionally, race/ethnicity, country of birth, and geographic region significantly influence dietary habits, further underscoring the complex interplay between demographic factors and food choices (53).

Another significant contributor to UPF consumption is the aggressive marketing strategies employed by food manufacturers. Extensive advertising through television, digital media, social platforms, and product packaging profoundly influences food choices, especially among younger consumers (3, 34). These marketing tactics are designed to exploit sensory and cognitive mechanisms, reinforcing the appeal of UPFs while often overriding awareness of their negative health consequences (34). Additionally, tradition, cultural influences, and individual psychological traits also shape dietary choices, contributing to varied dietary patterns across different populations (21). People often turn to UPFs as a coping mechanism for emotional distress, mistakenly believing that these foods provide relief from negative emotions. However, UPFs can impair self-control over food intake, reinforcing a cycle of emotional eating (45). This effect is particularly pronounced during adolescence, a critical period of vulnerability where emotional distress—especially among girls—can increase susceptibility to disordered eating behaviors (54).

## Brain regions involved in eating behaviors

Several brain areas, neuronal signals and physiological processes are involved in the regulation of eating behaviors. This control is mediated by two main regulatory systems: homeostatic and hedonic. Homeostatic control, stimulated by energy needs (i.e., hunger), responds to physiological signals from the periphery of the body. These signals are transmitted via nerve connections or as circulating endocrine hormones along the gut-brain axis (55, 56). In contrast, hedonic control is stimulated by a strong desire for appealing and tasty foods, primarily driven by pleasure and reward factors (57). Although these governing pathways are quite distinct, they share some overlapping components in the brain regions responsible for reward and decision-making, and most importantly they interact to regulate the search for food reward in response to physiological eating states (35, 56, 57).

The shared biological pathways between food reward and neural networks concerning other behaviors signal through dopaminergic neurons in the ventral tegmental area (VTA), which are fundamental to several food-related neuropsychiatric disorders and hedonic imbalances, including ADHD and autism spectrum disorder (ASD). Dysfunctions in the mesolimbic dopamine system have been linked to altered reward sensitivity and impulsive behavior in these conditions, which may be exacerbated by diets high in ultra-processed foods (58–60). Hedonic eating mechanisms, dominated by the reward system, allow the pleasurable features of food, along with the intensity and frequency of exposure to dietary cues, to override homeostatic signals (58). This shift can lead to excessive appetite stimulation, overconsumption, weight gain and the biological dysfunctions that underlie many eating disorders, such as obesity (54). Furthermore, while the hedonic properties of food, such as palatability, acts as appetite regulator by promoting eating, some studies suggest that taste itself does not seem to drive taste preferences and thus lead to long-term overconsumption. On the contrary, the homeostatic control of food intake, seems to be more focused on energy sources rather than sensory properties, appearing to be more decisive in influencing eating behavior (54, 61, 62).

The regulation of eating behavior is orchestrated by a network of cortical and subcortical brain regions, including the corticostriatal-limbic system, some regions of the brain stem, the hypothalamus and the thalamus. These areas integrate a variety of sensory, emotional and cognitive inputs to manage food intake. Specifically, the nucleus tractus solitarius (NTS) in the brain stem senses visceral cues through the vagus nerve. Concurrently, the lateral parabrachial nucleus (LPBN) processes these along with additional inputs from taste and olfactory receptors before relaying them to the central amygdala (63). This integration of post-prandial vagal signals plays a critical role in regulating the dopaminergic system, including the VTA, striatum and prefrontal cortex (PFC) (54). The hypothalamus integrates all the information related to body energy balance coming from the periphery (64). The hypothalamic arcuate nucleus (ARC) encloses neurons that respond to hunger and energy deprivation. Neurons expressing agouti-related protein (AgRP) stimulate appetite and motivate food consumption, whereas proopiomelanocortin (POMC) neurons suppress it (55, 63, 65). Adjacently, the paraventricular hypothalamic nucleus (PVN) cooperates with the ARC to integrate hunger and satiety signals delivered from NTS (63). The lateral hypothalamic area (LHA), through *γ*-aminobutyric acid (GABA) neuronal activity, functions as a feeding center by reinforcing food-related rewards, while the ventromedial hypothalamus serves as a satiety center (66, 67).

The corticostriatal-limbic system, which includes the PFC, hippocampus, amygdala, and the striatum, is pivotal in the higher level control of eating (54). The PFC, particularly, is responsible for the executive control, integrating information from various sources, including the hypothalamus and the limbic system (57). It acts as a final check, guided by attention, impulse control, learning, memory and cognitive flexibility, on eating decisions related to rewards and emotions (54). Research suggests that the ventromedial PFC drives food choices by evaluating sensory cues, rewards and assigning hedonic value to foods, while the lateral PFC may suppress the activity of the ventromedial PFC, helping to avoid unhealthy and tempting foods (64). Additionally, the developmental trajectory of the PFC may allow for several changes in reward perception over time (54).

Building upon the roles of the corticostriatal-limbic system, other brain regions also play significant roles in the regulation of eating. The hippocampus, crucial for forming and retrieving memories, influences eating behavior by recalling past food experiences, which may affect current food choices. Simultaneously, the amygdala assigns emotional significance to these eating experiences further influencing our reactions to different foods (64). Within the mesolimbic system, the striatum is essential for its role in regulating reward and promoting motivation, thereby reinforcing food-seeking behaviors (57). Similarly, the insular cortex plays a comprehensive role by integrating sensory cues with limbic inputs to generate feelings that ranges from satiety and hunger to thirst and even nausea (68, 69). This integration includes encoding the caloric content of food, a process influenced by intestinal hormone changes as well as cognitive expectations, such as anticipated taste (70, 71).

Finally, the gustatory thalamus and primary gustatory cortex process organoleptic properties of food, such as taste, texture and smell, and contribute to feeding behaviors (72). These areas are integral to the direct contributions to feeding behaviors, rounding out the complex network of brain regions involved in the regulation of eating.

## Key stages of brain development

Understanding and analyzing the development of the neuronal circuits involved in eating behavior, as well as in executive and cognitive functions, presents significant challenges. This complexity stems partly from the prolonged maturation of the brain, particularly the PFC, which continues to develop until around the age of 25 (73, 74). However, research has identified certain critical periods during brain development where external factors, such as diet, may have a more pronounced impact (54, 74).

The prenatal period and childhood have been shown to be critical time windows for the brain. In particular, the third trimester of pregnancy has been recognized as being highly determinant, with the brain evolving from a simple, smooth structure into a more complex one (75). Beginning around the 24th gestational week, critical processes such as myelination, the formation of synapses and the development of key brain regions involved in fundamental cognitive and reward functions take place. Significant developmental strides in brain areas such as the hippocampus, visual and auditory cortices and the striatum undergo their major developmental progresses over the last trimester and continue into early neonatal life (75). During these stages, the plastic properties of the fetal brain make it particularly vulnerable to structural and functional changes in response to maternal health behaviors, emphasizing the pivotal role of maternal diet. Nutrient deficiencies during this time can have lasting effects on the development of the child (13). As infants reach about 6 months old, they begin to develop and shape their taste preferences and eating habits, indicating the importance of diet in early life (76). The dietary patterns established during these early months can be predictive of future dietary behaviors, underscoring the long-term influence of early nutritional experiences (35).

As the discussion of early brain development moves to later stages of growth, it is important to note that developmental transitions during adolescence also signify critical changes, particularly in hedonic neuronal circuits (77). During this period, the PFC continuous to develop, including the growth of dopaminergic neurons that mediate communication with the striatum. Throughout this period, the activity of these neurons is particularly susceptible to alterations caused by external factors that can affect memory, inhibitory control, affective and reward processing. Notably, changes in the dopaminergic system during this developmental phase are associated with an increase of brain’s plasticity, enhancing its ability to adapt in response to experiences. Consequently, the adolescent brain becomes hyperresponsive, characterized by heightened activation of reward centers and increased sensitivity to rewarding cues (78, 79).

This strong sensory processing observed in adolescents can be explained through learning mechanism associated with food consumption. The experience of eating, particularly of highly palatable UPFs, is reinforced by the associated rewards, integrating emotional and cognitive processes, such as pleasure, motivation and learning. As a result, the cerebral reward circuitry may promote overconsumption in response to an excess of these stimuli from these foods, with the learning capacity of the reward system aiding memorization of the pleasurable sensations associated with UPFs, thereby encouraging repetition of experience (35). In addition, the ongoing development of dopamine neurons during childhood and adolescence marks these periods as particularly vulnerable to challenges in the regulation of emotions. During these times, individuals begin to develop emotional self-regulation strategies that improve throughout life, influenced by learning and individual temperament (69). Cognitive reappraisal is one of these strategies, consisting of changing the emotional impact and reinterpreting the emotional situation, and involves several brain areas that are also integral to food reward networks. This overlap explains the close relationship between emotional and eating processes that often underlies eating disorders such as binge eating disorder, which are particularly prevalent among young people (69).

Given the vulnerabilities in brain development during childhood and adolescence, the increasing consumption of UPFs among younger populations raises significant questions regarding long-term metabolic and behavioral consequences. Poor dietary habits in childhood are a modifiable risk factor for noncommunicable diseases, including obesity, metabolic disorders, and cardiovascular conditions (49). Over the past few decades, childhood and adolescent obesity rates have risen dramatically, increasing from 0.7 to 5.6% in boys and from 0.9 to 7.8% in girls between 1975 and 2016 (80). The most rapid weight gain occurs between ages 2 and 6, and studies indicate that 90% of children classified as obese at age 3 remain overweight or obese through adolescence (81). These trends highlight the need to address early dietary influences and their lasting health consequences.

Obesity is driven by a complex interaction of biological, socioeconomic, and environmental factors, with UPFs playing a central role (82). Their widespread availability, affordability, and hyperpalatable nature contribute to an obesogenic environment, in which dietary habits, sedentary behavior, and food marketing shape weight-related outcomes. UPFs, often high in added sugars, unhealthy fats, and refined carbohydrates, have been directly linked to poor satiety regulation, metabolic dysfunction, and excessive caloric intake (83, 84).

High screen time further exacerbates the issue by increasing exposure to food marketing, encouraging mindless eating, and reducing physical activity and sleep quality (83–85). Studies show that children consume more energy-dense foods during or shortly after viewing advertisements, reinforcing unhealthy eating behaviors from an early age (30). This cycle of early UPF consumption and reinforcement of food preferences plays a pivotal role in shaping long-term dietary behaviors. If UPFs dominate a child’s diet, their preference for sweet and salty foods is likely to persist into adulthood, reinforcing poor nutritional habits (29, 86, 87). Early exposure to sugar-sweetened beverages and UPFs has been linked to higher BMI, increased body fat percentage, and a greater likelihood of obesity in later life (45, 49). Additionally, early dietary experiences shape long-term food preferences—frequent consumption of sugary and processed foods in childhood fosters a preference for sweet and highly palatable foods in adulthood, potentially reducing the intake of nutrient-dense, health-promoting options (49, 88).

Beyond weight gain, early-life UPF exposure increases the risk of metabolic, inflammatory, and endocrine dysfunctions, predisposing children to chronic health complications later in life (89, 90). The interplay between brain development, food reward mechanisms, and metabolic health highlights the importance of addressing UPF consumption early in life to mitigate both cognitive and physiological consequences.

## Maternal UPF exposure and fetal neurodevelopmental changes

Transitioning from the impact of diet on adolescent brain development, it is equally crucial to consider how maternal nutrition during pregnancy influences fetal development. A recent study showed that UPFs constitute 17.2% of the total food intake among women in their third trimester of pregnancy (13). The period from 24 to 42 weeks of gestation represents a critical window for child neurodevelopment, during which processes such as synapse formation and myelination are actively taking place (75). The fetal brain is particularly plastic during this time and is susceptible to structural and functional changes due to maternal health characteristics, with potential long-term cognitive implications. Given the critical role of maternal nutrition in shaping perinatal health outcomes and long-term developmental trajectories of the offspring (91), a closer examination of UPF consumption is warranted to better understand its potential effects on fetal neuronal development.

Brain development is highly sensitive to environmental influences, as it involves a precisely coordinated sequence of critical processes that occur at specific developmental time points. Disruption of these stages—such as cell proliferation, neuronal migration, neurite outgrowth, and synapse formation—has been linked to neurodevelopmental disorders (92). Harmful environmental exposures during these critical developmental windows can exert profound effects on long-term mental health outcomes, consistent with the concept of the “developmental origins of health and disease (DOHaD)” (93, 94). The DOHaD, or the “Barker hypothesis,” postulates that adverse events during gestation or early postnatal life can permanently alter the structure and function of cells, tissues, and organs, thereby predisposing individuals to a range of health conditions, including behavioral and cognitive disorders. The maternal diet is a critical determinant of perinatal health, influencing the risk of complications such as gestational diabetes, hypertensive disorders, premature birth, and abnormal birth weight (95). Moreover, poor dietary patterns during pregnancy have been associated with an increased likelihood of developing chronic diseases later in life, including obesity, cardiovascular disorders, and type 2 diabetes mellitus (96). Despite the well-documented importance of maternal nutrition, research shows that many pregnant women consume high levels of UPFs, often at the expense of nutrient-dense, whole foods, which can negatively impact both maternal and fetal health (12, 14, 97). Research has indicated a notable link between diets high in UPFs during pregnancy and adverse outcomes, including excessive gestational weight gain (14), an increased risk of gestational diabetes mellitus (98), hypertensive disorders such as preeclampsia (99), low birth weight (100), and preterm birth (101). However, some studies have found no significant association between UPF consumption and these outcomes (11, 15).

During pregnancy, the body undergoes significant metabolic and physiological changes to support fetal growth and development, emphasizing the need for a nutrient-rich diet. Several biological mechanisms—such as inflammation, epigenetic changes, and alterations in the intestinal microbiome—have been identified as pathways through which maternal nutrition can influence offspring health outcomes (20). One key pathway is the gut-brain axis, a bidirectional communication system linking the gastrointestinal tract and the central nervous system. This axis plays a crucial role in regulating neurodevelopment, behavior, and immune responses, and is strongly influenced by maternal diet and gut microbiota composition (102).

The early-life environment is largely shaped by factors affecting the maternal environment, subsequently impacting the developing fetus or infant (103). Combined with genetic predispositions, these environmental influences—including maternal UPF consumption—can shift the balance toward or exacerbate the phenotypic manifestation of a disease initiated by a genetic insult. For instance, epigenetic changes, such as DNA methylation or histone modifications, can alter gene expression and initiate fetal programming processes that may increase the risk for neurodevelopmental disorders, including ASD (104, 105). Furthermore, maternal dietary patterns high in processed foods can significantly alter the composition and diversity of the infant’s gut microbiome (91, 106). This disruption can lead to increased inflammation, potentially influencing both fetal immune function and brain development (105, 107). Therefore, optimal nutrient intake before and during pregnancy is crucial, as deficiencies or excesses in micro-and macronutrients can activate fetal programming effects that persist into later life (105). Nutrient intake before and during pregnancy is paramount. Deficiencies or excess in micro-and macronutrients can activate fetal programming that may persist into later life (105).

Certain nutrients, such as long-chain polyunsaturated fatty acids, choline, protein, iron, and zinc, are particularly vital (75, 108–110). They support synaptic transmission in the developing fetal brain, and an imbalance or deficiency in these nutrients due to a poor maternal diet can lead to significant cognitive impairments in children (13, 111). Building on the importance of a balanced maternal diet, it is essential to understand that although all nutrients contribute to ensure to healthy fetal growth and neuronal development, some of them appear to be even more crucial during the previously mentioned vulnerable critical time windows. A deficiency in any of these nutrients can lead to severe cognitive impairments in the child. The fetal brain relies on nutrients such as long-chain polyunsaturated fatty acids, choline, protein, iron and zinc for efficient synaptic transmission. A deficiency in any of these nutrients in the maternal diet can result in severe cognitive impairments in the child (12). For this reason, these nutrient deficiencies have a strong impact, as they affect neuroanatomy, neurochemistry and neurophysiology, resulting in an altered global neuronal performance. If such deficiencies extend beyond periods of potential neurological repair, the damage can become irreversible (6).

For instance, a reduced protein intake during pregnancy can impair cognitive and verbal functions due to its effect on the hippocampus and cerebral cortex (112). Similarly, fetal iron deficiency causes changes in myelination, neurotransmitter synthesis and concentration particularly glutamate and striatal dopamine, and disrupts energy metabolism in the hippocampus and frontal cortex (113–115). Zinc deficiency plays an important role in the presynaptic release of neurotransmitters and is associated with reduced brain mass in critical areas such as the cerebellum, limbic system and cortex (116) and is linked to reduced electrophysiological activity, short-term memory and impaired development of the temporal and frontal lobes as well cerebellum in animal studies (117, 118). In addition, newborns of mothers with reduced zinc intake have shown alterations in the function of the hippocampus and in the regulation of the autonomic nervous system (117, 119). Moreover, long-chain polyunsaturated fatty acids are vital neuromodulators that influence synaptogenesis, membrane structure and function, supporting retinal and cognitive development, while their absence can result in significant developmental defects in the newborn (119). Notably, the last few months of pregnancy are particularly fragile, and a high maternal intake of saturated fats can increase oxidative stress, leading to neuroinflammation and potentially negative effects on the child’s cognitive functions (13). The placenta also plays a pivotal role in the developmental programming of the fetus and its brain, acting as the metabolic exchange between the mother and her child (120). Animal studies have linked placental insufficiency or intrauterine infection to impaired astrocyte development, microglial activation, white matter and blood–brain barrier damage in the offspring (120). A high fat maternal diet has been shown to increase placental inflammation, oxidative stress, altered neurotransmitter synthesis and lipotoxicity, all of which negatively affect fetal neurodevelopment (120, 121).

Recent studies further highlighted that maternal diet affects hedonic regulation of the growing fetus shaping food preferences that extend into childhood (69). An elevated maternal UPF consumption can profoundly impact the infant’s future food choices, reinforcing the critical need for optimal maternal nutrition during pregnancy (35).

Understanding the mechanisms linking maternal diet to fetal brain development is crucial. This review aims to further explore the impact of industrial foods on neurodevelopment. Maternal diet significantly shapes the mesocorticolimbic reward networks of offspring. For instance, mouse models have shown that a high-fat maternal diet can alter the density of hypothalamic projections, thereby affecting the development of the LHA (122). Additionally, research has demonstrated that the balance of omega-6 to omega-3 polyunsaturated fatty acids (PUFAs) during pregnancy can modulate hedonic circuits, thereby influencing a child’s preference for palatable foods (123). UPFs generate powerful rewarding stimuli influencing neuronal pathways promoting feeding. Activation of the reward system occurs through sensory stimuli, such as visual, olfactory or gustatory, as well as through internal signals. The organoleptic properties of UPFs act as reward signals, while the composition of refined carbohydrates and lipids causes rapid glycemic spikes and stimulation of the vagus nerve, triggering dopamine release (35). However, the impact of UPFs on brain development extends beyond reward mechanisms. A pivotal population-based birth cohort study firstly assessed the relationship between maternal UPF consumption during pregnancy and child neurodevelopment, using two standardized psychometric scales (13). The results showed a reduction in global cognitive functions, verbal expression, concept reasoning and memory in preschool children correlating with increasing maternal UPF intake during pregnancy. A weaker association was observed between maternal UPF consumption and perceptional performance, numeric and executive functions (13).

A Brazilian study suggests that UPF consumption in childhood predicts hyperactivity and inattention symptoms in later adolescence. This study also revealed that chronic consumption of foods high in refined sugars and saturated fats may alter dopamine activity, reducing cortical function and contributing to the expression of ADHD symptoms (36). Conversely, diets rich in fiber, folate and omega-3 fatty acids may have protective effects against the development of ADHD (36).

Building on the understanding of UPFs’ influence on neurodevelopmental outcomes, it is essential to examine how these foods compromise brain health through multiple mechanisms. UPFs promote neuroinflammation and alter neuronal communication and brain development by compromising the integrity of the blood–brain barrier and increasing its permeability. This increased permeability occurs due to changes in tight junction proteins and the activation of cytokine-producing microglia and astrocytes (34). Moreover, additives in UPFs, such as nanoparticles, can cross the blood–brain barrier (124). For example, titanium dioxide (TiO2) accumulates in glial cells and neurons, impacting memory, learning and locomotion, while silver nanoparticles accumulate in the brain causing short-and long-term memory impairment (34). UPFs also contribute to increased exposure to bisphenols, which can cross the placental barrier and disrupt ongoing fetal brain development. These chemicals interfere with genes involved in dopamine and serotonin neurotransmission, potentially leading to later behavioral disorders, such as anxiety and hyperactivity later in life (34). During development bisphenols may further impact critical brain areas such as the hypothalamus, amygdala and hippocampus, and in adults, they may persist in the bloodstream for extended periods and potentially damage brain tissues (34). Furthermore, trans fats found in UPFs can alter the composition of brain membrane phospholipids, impairing neuronal communication (34). Studies in rats have shown that consumption of trans fats during pregnancy and lactation leads to an increase in oxidative stress and pro-inflammatory cytokines in the child’s brain, particularly in the hippocampus and cortex, affecting memory and anxiety behavior (34). These findings underscore the pervasive impact of UPFs on brain health, affecting not just developing brains but also adult brain function and structural integrity.

## Adolescent and adult UPF consumption and neurocognitive consequences

Adolescence is a second “critical window” for brain plasticity. Heightened ventral-striatal reactivity together with still-maturing pre-frontal control makes teenagers exceptionally sensitive to reward cues (77, 78). In a cohort of 6,380 European youths, every 10 % increase in daily UPF energy predicted a 0.11 SD decrement in composite executive-function scores independent of adiposity and socioeconomic status (36). Experimental work shows that the fat-plus-sugar combinations typical of UPFs evoke supra-additive mid-brain dopamine firing, reinforcing cue-triggered “wanting” and accelerating the shift from goal-directed to habitual intake (125).

In adults, chronic UPF exposure is associated with structural and functional brain changes that precede clinical neurodegeneration. Longitudinal data from the Raine Study link high-UPF diets to a 5 % reduction in hippocampal volume after adjustment for vascular risk factors (126). Two complementary datasets, the 2025 Framingham analysis and a 2024 meta-analysis of nine cohorts, show a 25–35% excess risk of all-cause dementia in the highest UPF quintile (127, 128). Mechanistically, additive-rich, fiber-poor formulations foster gut dysbiosis, systemic inflammation and insulin resistance, all of which potentiate hippocampal shrinkage and disrupt fronto-striatal connectivity (129).

Dietary habits established in early life shape future behaviors and contribute to long-term health outcomes, including the risk of serious diseases later in life (35). During critical developmental periods such as childhood and adolescence, the brain undergoes significant structural and functional changes, making it particularly vulnerable to external influences, including diet (54). These dietary impacts are not limited to function but also affect brain morphology. For example, Jacka and colleagues found that greater adherence to a Western-style diet high in processed foods was associated with a smaller hippocampal volume, while a healthier “prudent” diet correlated with larger hippocampal size (126). When assessing food, the brain assigns a subjective value based on characteristics such as taste, flavor, and energy content. This evaluation involves interactions between sensory and emotional stimuli, as well as executive signals from different brain regions, converging in the ventromedial PFC (130).

The composition and texture of UPFs play a crucial role in food choices, consumption patterns, and overall energy intake (125). UPFs, which are typically energy-dense and soft in texture, accelerate eating rates and reduce exposure to orosensory cues, thereby decreasing satiety and promoting overconsumption (131, 132). Additionally, the sensory homogeneity of these foods, their consistent texture, flavor, and appearance, may reinforce rigid eating patterns in certain individuals. This is particularly relevant in the context of Avoidant/Restrictive Food Intake Disorder (ARFID), a feeding and eating disorder characterized by extreme selectivity or avoidance of specific foods due to sensory sensitivities, fear of adverse consequences, or lack of interest in eating. Individuals with ARFID often limit their diets to a narrow range of UPFs that meet their sensory preferences, which may exacerbate nutritional deficiencies and impair growth or cognitive development, especially in children and adolescents (133). Emerging research suggests that ARFID is not only more common than previously recognized but may be exacerbated by the overabundance of uniform, palatable processed foods in the modern diet. These products may unintentionally reinforce food avoidant behaviors by making it easier to maintain a highly selective intake pattern without immediate satiety or nutritional feedback (133).

A randomized controlled trial found that meal-eating rates were higher when participants followed an ultra-processed diet, suggesting that these foods may delay satiety signals and thus increase energy intake due to overeating (2). Furthermore, frequent consumption of saturated fats, refined sugars, and salt has been shown to alter taste perception, shifting preferences toward highly processed foods, thereby reinforcing habitual consumption patterns through chemosensory plasticity (34, 69).

UPFs, which often contain a combination of refined sugars and unhealthy fats, intensely stimulate the brain’s striatal reward system, increasing the likelihood of habitual overconsumption (130). Studies suggest that frequent consumption of foods high in sugar alters key brain regions involved in eating behavior, leading to decreased striatal and dopaminergic reward responses, contributing to habit-driven eating patterns and reduced sensory satiety (35). Additionally, low-and no-calorie sweeteners do not activate brain regions responsible for appetite, such as the hypothalamus and insula, but do activate reward-related areas such as the nucleus accumbens, potentially prolonging meal duration, increasing energy intake, and disrupting satiety signaling (134). These findings support the theory that eating behaviors and food choices are more strongly influenced by energy content than by sensory properties alone (61).

Since energetic signals from food activate the brain’s reward system via dopaminergic neurons, the potential addictive properties of highly palatable foods remain a topic of ongoing debate among researchers (54, 69, 135). Dysfunction in dopamine and serotonin circuits, key neurotransmitters involved in reinforcement, motivation, mood, and cognition, has been associated with unhealthy dietary habits and eating disorders. Chronic UPF consumption has been linked to inflammatory processes in the brain, oxidative stress, and neurodegenerative diseases, contributing to cognitive decline, depression, and potentially an increased risk for Alzheimer’s disease (127, 128). However, the impact of UPFs on brain function is not limited to direct neural mechanisms—it is also mediated through the gut-brain axis, a bidirectional communication system between the gastrointestinal tract and the brain that plays a crucial role in energy regulation, mood, and cognitive function.

## Gut-brain interaction: a key pathway linking UPFs to cognitive and metabolic dysfunction

The gut-brain axis plays a central role in regulating homeostatic and reward-driven eating behaviors by integrating signals between the digestive system, hypothalamus, and brainstem (54). This communication network modulates dopamine signaling in reward circuits, reinforcing food preferences based on the metabolic value of ingested nutrients (135). Reinforcement occurs when metabolic signals released in the intestine upon nutrient absorption activate the brain’s reward pathways, enhancing motivation for high-energy foods, often independently of conscious perception (135). However, processed foods characterized by higher energy density, refined carbohydrates, and artificial additives interfere with and impair gut-brain communication (54). These disruptions are associated with increased cravings and compulsive eating behaviors, heightened reward-system activation, and weakened satiety signals, ultimately contributing to excessive energy intake and long-term metabolic dysregulation (2, 54, 135).

Beyond its role in nutrient signaling, the gut microbiome is a critical regulator of hunger and satiety and significantly impacts neurochemical processes involved in emotional and cognitive function (129, 136). It plays a key role in the production of serotonin, a neurotransmitter that influences mood, appetite, and cognition with approximately 90% of the body’s serotonin synthesized in the gut (137). Additionally, the gut microbiota influences levels of brain-derived neurotrophic factor (BDNF), a protein essential for neuroplasticity, learning, and memory. Altered BDNF signaling has been linked to mood disorders and impaired cognitive development. Disruptions in gut health caused by diets high in ultra-processed foods can reduce both serotonin availability and BDNF expression, thereby negatively affecting both emotional regulation and brain function (138).

The microbiome influences the secretion of intestinal hormones, modifies neurotransmitter metabolism, and synthesizes short-chain fatty acids (SCFAs) from dietary fiber fermentation, which can cross the blood–brain barrier and regulate energy homeostasis and brain function (136). However, diets low in fiber and high in saturated fats and refined sugars alter microbiome diversity and composition, disrupting gut-brain signaling and negatively affecting neurochemistry (139). UPF-induced gut dysbiosis has been linked to increased intestinal permeability, allowing inflammatory molecules and bacterial endotoxins to enter systemic circulation, triggering neuroinflammation and oxidative stress, which may contribute to mood disorders, such as depression and anxiety (139, 140).

Emerging research suggests that the gut microbiota also plays a key role in neurodevelopmental disorders, particularly ADHD and ASD (36, 105). Maternal gut dysbiosis, often induced by high-UPF diets, reduces essential neuroactive metabolites, such as SCFAs and branched-chain amino acids (BCAAs), which are vital for neuronal development (105). Additionally, alterations in the maternal microbiome may interfere with the proper maturation of the enteric nervous system (ENS), a neural network that mediates interactions between the gut and the central nervous system (CNS). Since the ENS develops postnatally in parallel with microbial colonization, disruptions in maternal diet and microbiome balance may have long-term consequences for gut-brain communication (105).

Another concern is the increased exposure to food additives and contaminants found in UPFs, which may exacerbate gut-brain dysfunction. Studies have shown that nanoparticles, such as titanium dioxide (TiO2) and silver nanoparticles, cross the blood–brain barrier, accumulating in neurons and glial cells, where they impair memory, learning, and locomotion (124). Additionally, bisphenols, commonly found in food packaging, can disrupt fetal brain development by altering dopamine and serotonin neurotransmission, which may contribute to later behavioral disorders, including anxiety and hyperactivity (71).

The interplay between diet, gut health, and brain function highlights the importance of maintaining a balanced, fiber-rich diet to promote microbiome diversity, neuroprotection, and mental well-being throughout life. These findings highlight increasing awareness of the neurological consequences of prolonged UPF consumption, emphasizing the need for dietary interventions that support both gut health and cognitive function.

## Conclusions and future directions

**Figure 1:**
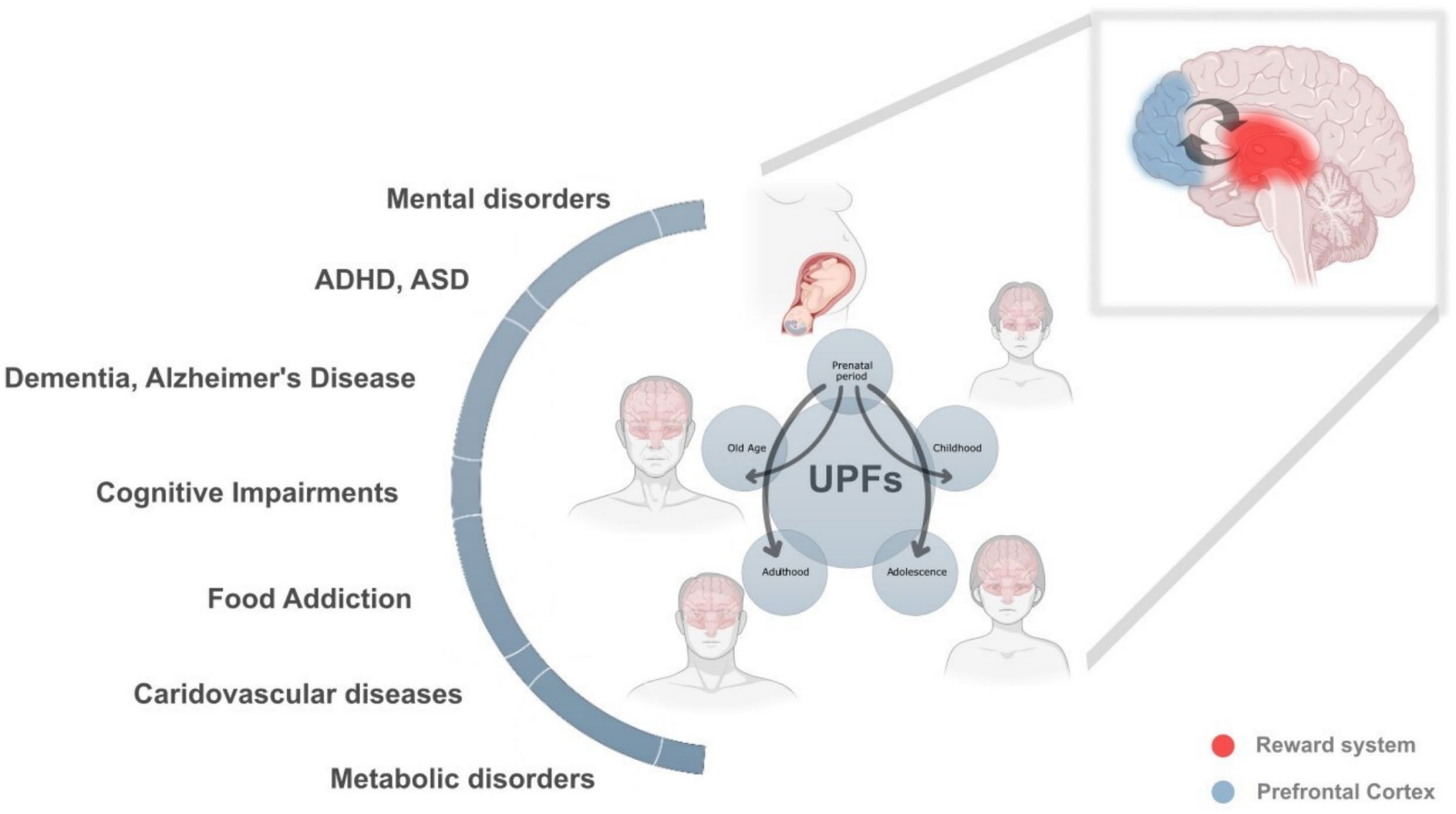
The lifelong and intergenerational impact of ultra-processed foods (UPFs) on health and neurodevelopment. This figure illustrates the profound and cumulative effects of UPF consumption across different life stages, prenatal period, childhood, adolescence, adulthood, and old age, highlighting their role in a broad spectrum of neurodevelopmental, metabolic, cardiovascular, and cognitive disorders. The interconnected arrows emphasize how exposure to UPFs in one stage can amplify health risks in later stages, creating a continuous and reinforcing cycle of adverse health outcomes. At each stage of life, UPF consumption can contribute to multiple health conditions, including: • Neurodevelopmental disorders (attention-deficit hyperactivity disorder (ADHD), autism spectrum disorder (ASD), cognitive impairments). • Mental health disorders (anxiety, depression, eating disorders, addiction-like behaviors). • Metabolic and cardiovascular diseases (obesity, diabetes, cardiovascular disorders). • Neurodegenerative diseases (Alzheimer’s disease, dementia). Lifelong health trajectories and UPFs **Prenatal period:** Maternal UPF consumption alters fetal brain development, particularly in the prefrontal cortex (blue) and the reward system (red), predisposing the offspring to cognitive impairments, behavioral disorders, metabolic dysfunction, and increased susceptibility to hedonic eating patterns. These early disruptions lay the foundation for long-term health risks. **Childhood:** A diet high in UPFs during early life further disrupts neurocognitive development, contributing to ADHD, food addiction, emotional dysregulation, and metabolic imbalances. These effects set the stage for difficulties in learning, impulse control, and emotional stability. **Adolescence:** This critical phase of brain maturation is particularly vulnerable to the addictive properties of UPFs, reinforcing unhealthy eating patterns and impairing decision-making and impulse control. Mental disorders, cognitive decline, and metabolic conditions often emerge or worsen at this stage. **Adulthood:** Prolonged exposure to UPFs leads to chronic inflammation, insulin resistance, cardiovascular diseases, and an increased risk of neurodegenerative disorders. These effects are compounded by previous life-stage exposures, reinforcing a cycle of poor health outcomes. **Old age:** The cumulative damage from lifelong UPF consumption accelerates brain aging, cognitive decline, and dementia, with increased susceptibility to Alzheimer’s disease and other neurodegenerative disorders. Metabolic and cardiovascular diseases further exacerbate age-related deterioration. The vicious cycle of intergenerational health adversities This figure also highlights how maternal UPF consumption initiates a vicious cycle that extends across generations. Poor maternal nutrition predisposes offspring to metabolic and neurological vulnerabilities, making them more susceptible to the “second hit” of their own UPF consumption later in life. These epigenetic and developmental disruptions, rather than genetics alone, explain the rising prevalence of obesity, neurodegenerative disorders, cognitive impairments, and motivational/hedonic eating behaviors across successive generations.

Ultra-processed foods jeopardise brain health across the life-course, as summarised in Figure 1. Evidence now links prenatal, childhood, adolescent and adult exposure to a continuum of neurocognitive harm that ranges from executive-function deficits and reward-circuit dysregulation to dementia. Key mechanistic threads include dopaminergic hypersensitisation, hippocampal vulnerability to metabolic inflammation, and disruption of the gut–brain axis. Because these risks accumulate across time and generations, the greatest benefit will come from interventions that start early and minimise cumulative dose. Policy levers that curb UPF availability, require unambiguous front-of-pack labelling, and stimulate reformulation are urgently needed. Longitudinal neuro-imaging with objective dietary metrics should be prioritised to confirm causality and pinpoint sensitive windows. Meanwhile, clinicians can act now by helping patients substitute UPFs with minimally processed, fiber-rich foods whenever feasible.
